# Transcriptome Analysis Identifies the Dysregulation of Ultraviolet Target Genes in Human Skin Cancers

**DOI:** 10.1371/journal.pone.0163054

**Published:** 2016-09-19

**Authors:** Yao Shen, Arianna L. Kim, Rong Du, Liang Liu

**Affiliations:** 1 Department of Systems Biology, Columbia University, New York, NY, United States of America; 2 Department of Dermatology, Columbia University, New York, NY, United States of America; Ohio State University Wexner Medical Center, UNITED STATES

## Abstract

Exposure to ultraviolet radiation (UVR) is a major risk factor for both melanoma and non-melanoma skin cancers. In addition to its mutagenic effect, UVR can also induce substantial transcriptional instability in skin cells affecting thousands of genes, including many cancer genes, suggesting that transcriptional instability may be another important etiological factor in skin photocarcinogenesis. In this study, we performed detailed transcriptomic profiling studies to characterize the kinetic changes in global gene expression in human keratinocytes exposed to different UVR conditions. We identified a subset of UV-responsive genes as UV signature genes (UVSGs) based on 1) conserved UV-responsiveness of this subset of genes among different keratinocyte lines; and 2) UV-induced persistent changes in their mRNA levels long after exposure. Interestingly, 11 of the UVSGs were shown to be critical to skin cancer cell proliferation and survival. Through computational Gene Set Enrichment Analysis, we demonstrated that a significant portion of the UVSGs were dysregulated in human skin squamous cell carcinomas, but not in other human malignancies. This highlights the potential and specificity of the UVSGs in clinical diagnosis of UV damage and stratification of skin cancer risk.

## Introduction

Skin cancer is the most common cancer in the US and has become a major public health problem due to rising incidence and treatment costs [1, 2]. Every year nearly 5 million cases of skin cancers are treated, at an estimated cost of $8.1 billion [3]. There is compelling scientific and epidemiological evidence that skin cancer is caused mainly by an interplay between genetic factors and exposure to solar UV radiation (UVR) [4–6]. Most skin cancer cases are preventable through proper protection against excessive UV exposure. Sunscreen is one of the commonly used sun protection strategies, especially in skin cancer susceptible populations [7]. However, there are controversies surrounding the efficacy and safety of sunscreen products [8–11]. Currently, the sun-protection efficacy of sunscreens is measured by Minimal Erythema Dose (MED), which refers to the lowest time interval or dosage of UV sufficient to produce a minimal, perceptible erythema on unprotected skin. As an indicator of UV damage, MED is both insensitive and inadequate because significant molecular and cellular damage occurs at sub-erythema UV doses that may contribute to cumulative photo damage [12, 13]. MED also varies widely among different skin types. To reduce skin cancer incidence, sensitive biomarkers and methods are needed for accurate assessment of UV-induced damage and evaluation of the protective efficacy of sunscreen products to enhance skin cancer prevention.

Presently, no consensus panel of UV biomarkers is available for quantifying UV damage and stratifying skin cancer risk. Previous studies have attempted to identify UV-responsive genes as UV biomarkers [14–19]. However, there are significant variations in the experimental designs including the choices of cell types, UV sources and doses, time points of analysis, and expression profiling methods with different genomic coverage. Such variances make cross-comparison and validation of previous findings challenging. To define a consensus UV biomarker panel with broad applicability and accuracy, we performed RNA-Seq studies to generate a transcriptomic cohort containing UV-responsive genes in human skin cells exposed to different UVR conditions. We then performed rigorous bioinformatics analysis to define a UV gene expression signature that is conserved among cells from different donors. We further demonstrated that the UV signature genes (UVSGs) are significantly enriched in genes dysregulated in human skin squamous cell carcinomas (SCCs), highlighting the potential clinical utility of the UVSGs in risk prediction and stratification of skin cancer.

## Material and Methods

### Human keratinocyte cultures, human SCC and normal skin tissues

Primary human keratinocytes from neonatal foreskins were provided by the Columbia University Medical Center Skin Disease Research Center’s Tissue Culture and Histology Core facility. The protocol was exempt by the Institutional Review Board at Columbia University. Keratinocytes from 4 individual donors (N0, N1, N2, and N6) were cultured in 154CF medium supplemented with human keratinocyte growth supplement (Life Technologies, Grand Island, NY). Cells from each donor were maintained and analyzed separately. Human SCC tumor tissues and adjacent normal skin tissues were obtained from the Molecular Pathology Shared Resource/Tissue Bank of the Herbert Irving Comprehensive Cancer Center of Columbia University under CUMC IRB protocol AAAB2667.

### UV radiation

Primary keratinocytes were propagated in culture for 2 to 3 passages in 10 cm culture dishes. UVR was delivered using four FS20T12 UV tubes, which emit UV rays between 290 and 340 nm, with an emission peak at 310 nm [20]. Before UVR, cells were rinsed once with PBS and irradiated in approximately 0.5 ml PBS in each 10 cm dish with 3 different doses: 10, 20, or 30 mJ/cm^2^, which was determined by an IL1400 radiometer connected to a SEL240/UVB-1/TD detector [21]. Cells were collected at 4 h, 1 day or 3 days after UVR. The seeding density for each UVR condition was adjusted based on a pre-determined LD50 UV dose (35 mJ/cm^2^ at 24 h after UVR) as follows [22]: 0.75x10^6^/dish for control cells or cells irradiated with 10 mJ/cm^2^ UVR; 1x10^6^/dish for cells irradiated with 20 mJ/cm^2^ UVR; 1.5x10^6^/dish for cells irradiated with 30 mJ/cm^2^ UVR (collected 1 day after UVR) or 3x10^6^/dish for cells irradiated with 30 mJ/cm^2^ UV (collected 3 days after UVR). Cells were allowed to recover for 24 h after subculture. UVR was applied in the order of 3 days, 1 day, and 4 h prior to the collection of all cells on the same day to ensure that all cells were cultured for the same amount of time within each experiment.

### RNA isolation and RNA-Seq analysis

Total RNA was isolated from cultured keratinocytes, human SCC tissues, and adjacent normal skin tissues using the RNeasy Kit (QIAGEN, Gaithersburg, MD). All RNA samples were subsequently analyzed using an RNA 6000 nano chip (Agilent Technologies, Wilmington, DE) to confirm that the RNA integrity index was 8.0 or above. Total RNA (500 ng) from each sample was subjected to poly-A pull-down to enrich mRNAs for library preparation by using Illumina TruSeq RNA prep kit (Illumina, San Diego, CA). The resulting libraries were sequenced using Illumina HiSeq2000 at Columbia Genome Center. Sequencing reads were mapped to the human reference genome (NCBI/build37.2) using Tophat (version 2.0.4). Differentially expressed genes (DEGs) between each UVR-treated group and the control group were determined using the DESeq software package [23], which is an R Bioconductor package that defines DEGs based on sequence read counts data. DESeq uses a negative binomial model to approximate the distribution of read counts and estimate the mean and variance of the dispersion. We selected DEGs using fold change (FC) cutoffs set at >2 or <0.5 between irradiated and non-irradiated keratinocytes. A False Discovery rate (FDR) of <0.05 was used to control for false discoveries.

### Bioinformatics and statistical analyses

DEG lists were used in principal component analysis (PCA) to test the variations in transcriptomic response to different UVR conditions among the keratinocyte lines. PCA is a statistical method that performs orthogonal linear transformation to convert a gene expression matrix into a linearly uncorrelated variable called the principle component (PC) [24]. Each PC is a linear combination of the original gene expression values. The first two PCs contain the most variance in the data, and could be visualized in 2-dimentional space. DAVID is a free online bioinformatics resource for systematic analyses of gene function classification, annotation and clustering based on various databases including KEGG, BioCarta, and Gene Ontology [25]. We used DAVID to identify the biological pathways in which the UV-induced DEGs were enriched. To determine the overlap between UVSGs and SCC signature genes, we performed gene set enrichment analysis (GSEA), which computes the enrichment of a pre-defined gene set on a gene signature generated by fold-change, t-test or other method using the Kolmogorov-Smirnov statistic [26].

We used Paired t-test to identify genes displaying time-dependent UVR responses following exposure. To identify genes manifesting UV dose-dependent changes, we constructed a linear regression model using UV dose as an independent variable and gene expression as a dependent variable for each gene in the same keratinocyte line and at the same time point. To evaluate the overall effects of the various UVR doses on the expression of a specific gene, we integrated the multiple p-values from every regression analysis for that gene using Fisher's Method.

To obtain cancer-specific gene signatures for various human malignancies, we retrieved the RNA-Seq raw counts from The Cancer Genome Atlas (TCGA) database, which contains genomic and clinical information for tumors and their matching normal tissues across over 30 cancer types from more than 11,000 patients (publically available at cancergenome.nih.gov). We selected RNA-Seq data sets with both primary tumors and matched normal tissues for each tumor type. We generated DEG sets for each tumor type using the DESeq software package. To identify genes critical to skin cancer cell proliferation or survival, we queried the Achilles database with 67 of the UVSGs that were upregulated by UVR [27]. Genes were considered essential to skin cancer cell survival if their corresponding shRNAs became depleted after 40 days or 16 population doublings following shRNA infection. Normalized shRNA depletion scores were downloaded from the "cBOTv8_sbsv3_allreps_log.gct2” file in the Achilles database. All statistical analyses were performed using the R software package.

## Results

### Transcriptomic responses to different UVR conditions

**Fig 1:**
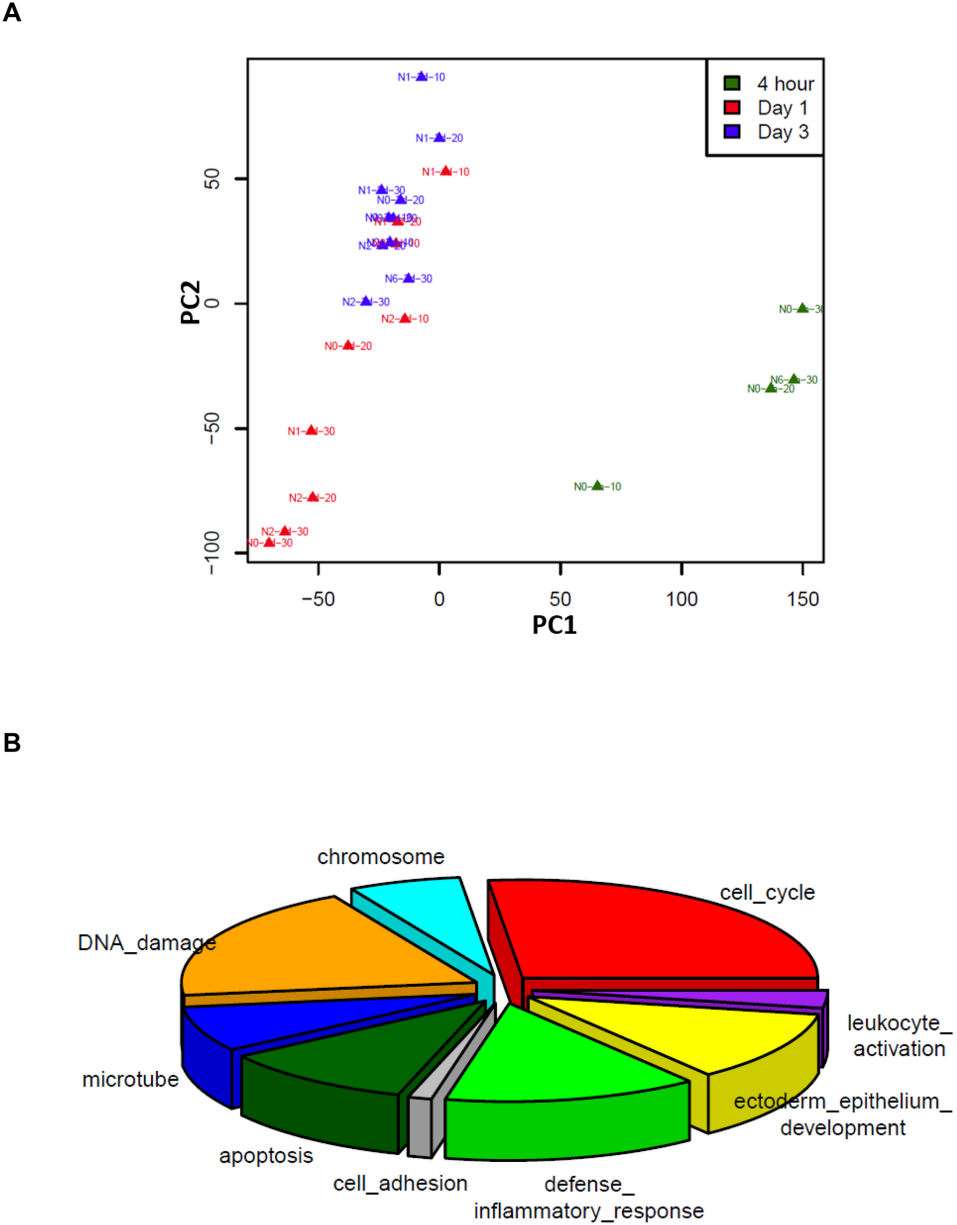
A: PCA analysis demonstrating time-dependent clustering of UV-responsive transcriptomic profiles in human keratinocytes; B: DAVID pathway analysis illustrating the enrichment of UV-induced DEGs into different biological pathways. The size of the pie chart is proportional to the number of genes in each pathway.

In addition to its mutagenic effect, UVR can cause genome-wide transcriptional instability affecting thousands of genes [14–16]. To fully characterize UVR-induced transcriptomic changes, we conducted RNA-Seq to profile mRNA expression changes in keratinocytes isolated from 4 individual donor foreskins in response to different UVR doses at varied time points after exposure. The resulting 22 DEG lists were subjected to principle component analysis (PCA) to visualize the similarities of the DEG profiles in response to different UVR conditions. As shown in Fig 1A, DEG profiles from Day 1 and 3 groups, but not the 4 h group, demonstrated great similarities in the first principle component (PC1). Along the second principle component (PC2) axis, the range of differences among the Day 3 DEG lists appeared smaller than that of the Day 1 DEG group, demonstrating a time-dependent increase in the similarity of UVR-induced transcriptomic changes among different keratinocyte lines and different UVR doses.

To uncover the biological pathways that were most responsive to UVR, we calculated the average of the FC of each DEG from Day 1 and Day 3 DEG lists (S1 Table). Using an FC cutoff of 2, we obtained a total of 531 genes that were upregulated (FC>2) and 610 genes that were down-regulated (FC<0.5) in response to different UVR conditions. We performed DAVID analysis to identify top biological pathways in which the upregulated genes and down-regulated genes were enriched. DAVID revealed multiple pathways that were significantly modulated by UVR. The down-regulated genes were significantly enriched in the following top biological pathways: cell cycle regulation (83 genes), chromosome structure (19 genes), DNA damage response (DDR) (59 genes), and microtubule organization (23 genes); whereas the upregulated genes were enriched in pathways such as apoptosis (33 genes), defense inflammatory response (43 genes), ectoderm epithelium development (36 genes), cell adhesion (4 genes) and leukocyte activation (9 genes) (Fig 1B).

### Time-dependent transcriptomic changes in response to UVR

**Fig 2:**
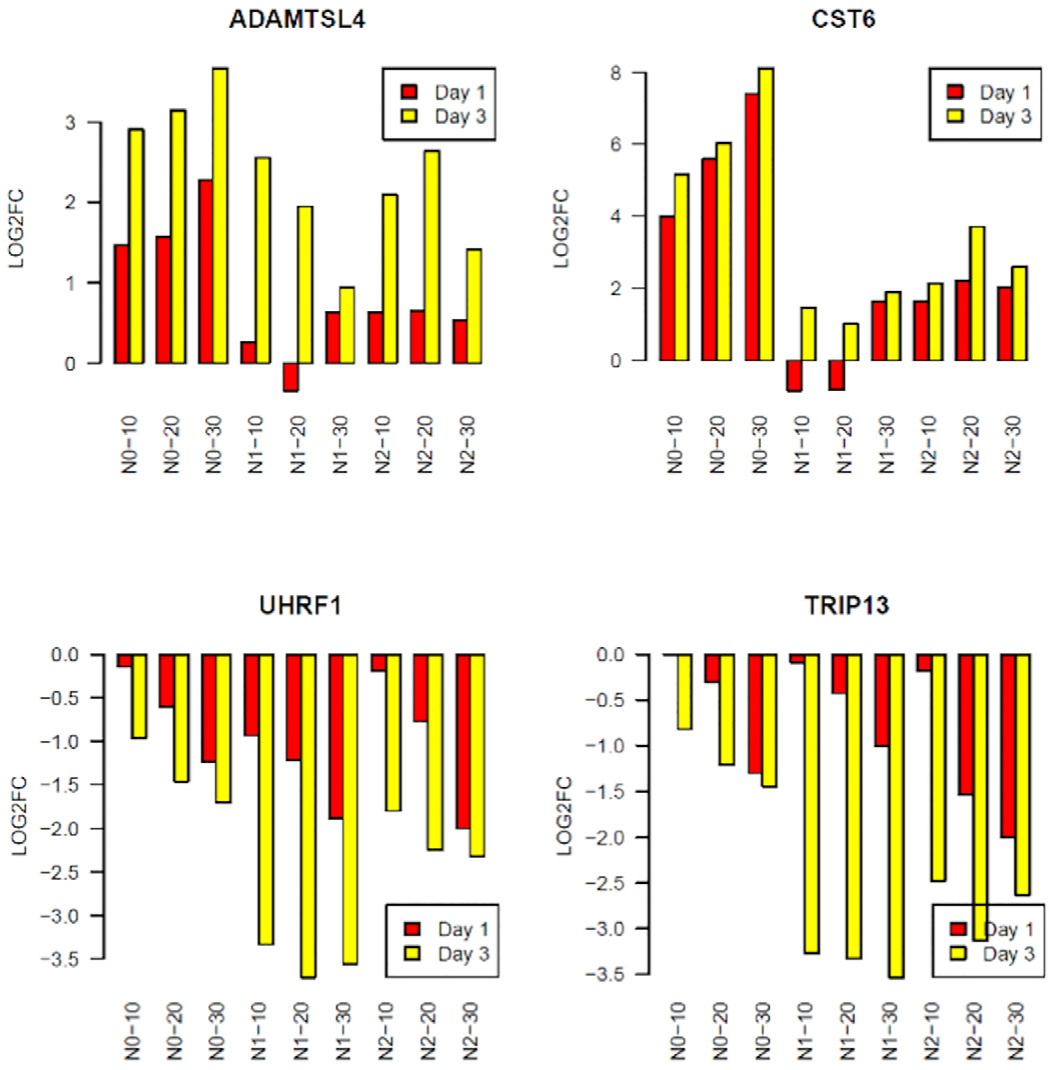
Graphs showing time-dependent changes in gene expression of UVR-target genes at Day 3 (yellow) and Day 1 (red). The x-axis represents the sample names. The y-axis shows the log2 fold change of gene expression between irradiated and non-irradiated control cells. ADAMTSL4 and CST6 showed time-dependent upregulation, while UHRF1 and TRIP13 displayed time-dependent down-regulation in response to UVR.

The PCA analysis in Fig 1A revealed time-dependent changes in the transcriptome following UVR. To identify which genes exhibit time-dependent UVR responses, we performed paired t-tests to compare the FCs of Day 3 DEGs with those of Day 1 DEGs for each keratinocyte cell line (N0, N1 and N2) under the same UVR dose. We found that 164 out of the 531 upregulated genes had higher expressions at Day 3 than at Day 1 (FDR-corrected p-value<0.05); while 239 out of the 610 down-regulated genes were more repressed at Day 3 than at Day 1 at the same p-value threshold (S2 Table). Two examples of time-dependent upregulation include ADAMTSL4, encoding a disintegrin and metalloproteinase; and CST6, encoding a cystatin superfamily protein. Examples of time-dependent down-regulation include UHRF1, encoding a member of a subfamily of RING-finger type E3 ubiquitin ligases; and TRIP13, which encodes a protein that interacts with thyroid hormone receptors (Fig 2).

### Dose-dependent transcriptomic changes in response to UVR

**Fig 3:**
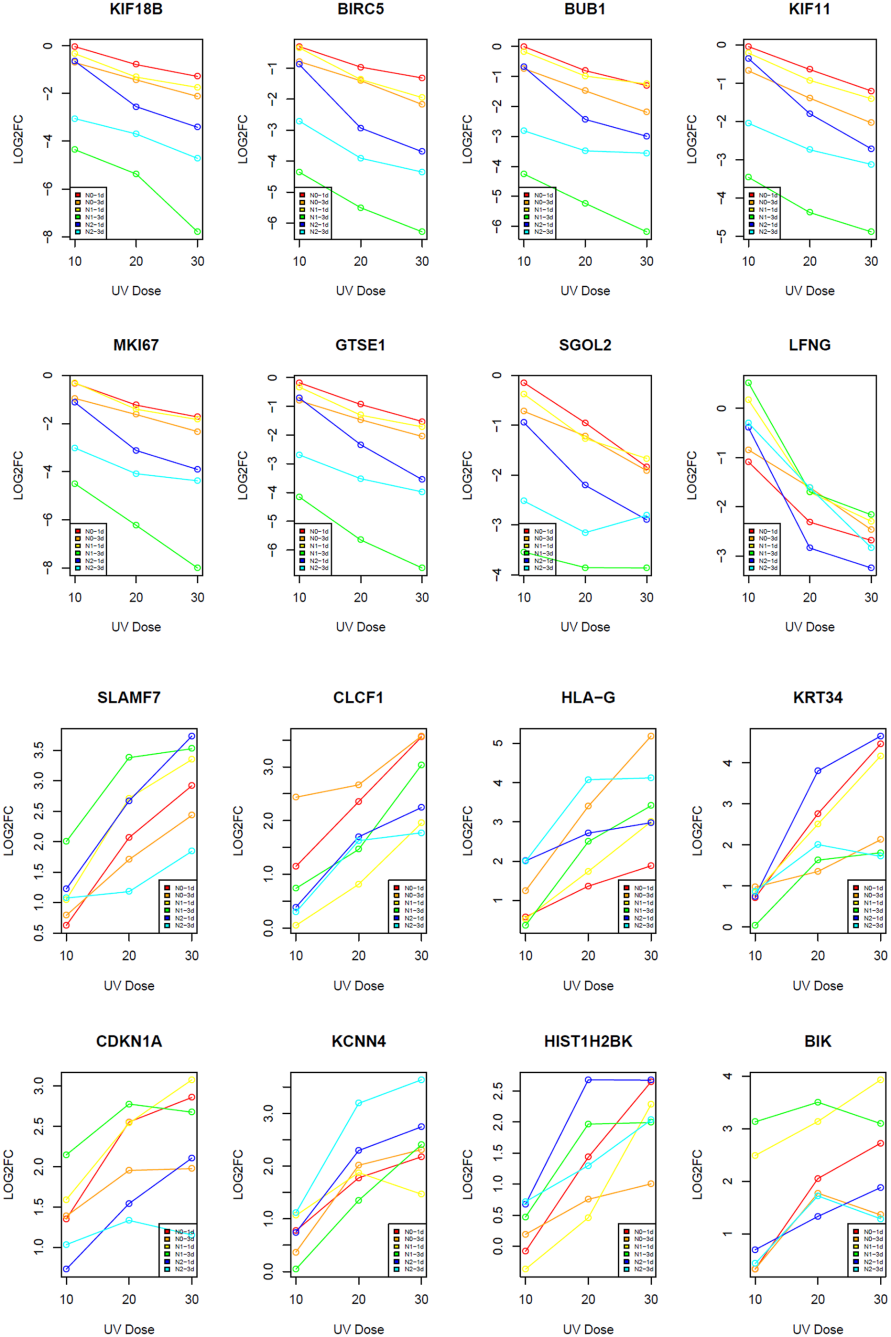
Plots showing dose-dependent down-regulation (upper two panels) and upregulation (lower two panels) of UVR-induced DEGs. Each point represents a sample at the corresponding UVR dose. X-axis represents three different UVR doses; Y-axis represents the log2 fold change of gene expression between irradiated and non-irradiated control cells. N0-1d, N0-3d, N1-1d, N1-3d, N2-1d, and N2-3d are delineated in red, orange, yellow, green, blue and cyan, respectively.

Next, we tested which genes displayed dose-dependent mRNA expression changes in response to UVR. To do so, we fitted linear regression models for each of the differentially expressed genes using UVR doses (10, 20 and 30 mJ/cm^2^) as independent variables and gene expression as the dependent variable for each keratinocyte cell line (N0, N1, N2) at the same time point (Day 1 or 3). For each gene, we constructed 6 models representing the following 6 conditions: N0-1d, N0-3d, N1-1d, N1-3d, N2-1d and N2-3d. We then integrated the 6 coefficient p-values from the 6 models using Fisher’s method. We found that 285 out of the 531 upregulated genes showed dose-dependent up-regulation with FDR-corrected p-value<0.05, and 452 out of the 610 down-regulated genes demonstrated significant dose-dependent decreases in gene expression at the same FDR threshold (S3 Table). Dose-dependent changes in 8 representative genes with the lowest p-values from each group are illustrated in Fig 3.

### Identification of conserved UVR transcriptomic signature genes

**Fig 4:**
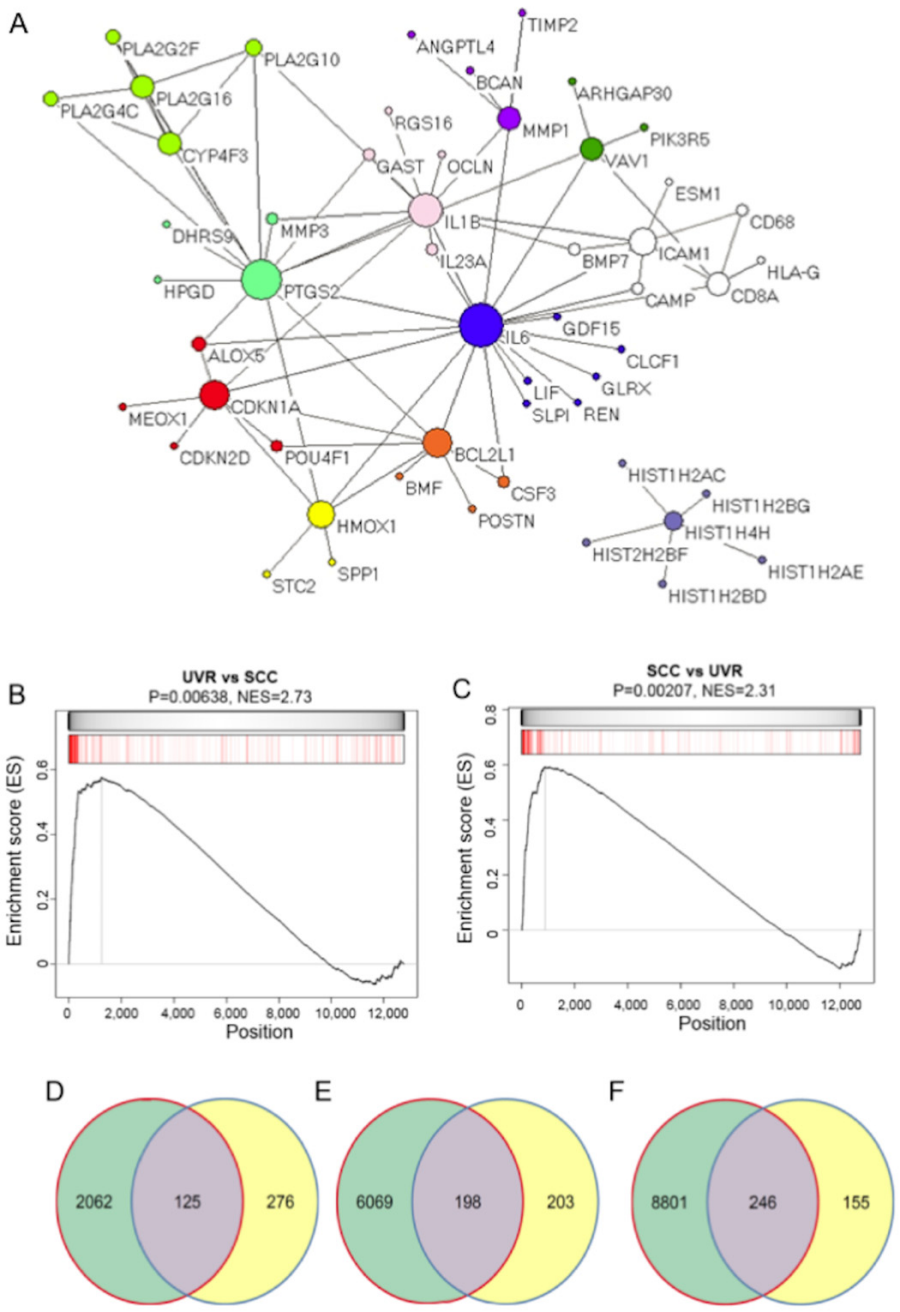
A: Protein-protein interaction network map illustrating hub genes as well as their interacting partners among UVSGs. Each vertice represents a gene in the PPI map and each edge indicates an interaction between the two genes. Genes belonging to different clusters are presented in different colors. The size of each vertice refers to the number of other genes that it is connected with, which is proportional to their degrees (number of interacting genes); B: GSEA of the genes dysregulated in human SCCs (red bars) against the UV signature. UVSGs were sorted from the highest (left end of the bar) to the lowest (right end of the bar) based on their FC. The normalized enrichment score (NES) and p value are indicated; C: GSEA of the UVSG set (red bars) against the SCC signature set. SCC signature genes were sorted from the highest (left end of the bar) to the lowest (right end of the bar) based on their FC; D: Venn diagram showing the overlap between UVSGs and DEGs at 21 days after UVR; E & F: Venn diagrams showing the overlap between UVSGs and DEGs from two SCC patients.

The time-dependent kinetic changes illustrated in Fig 2 suggested that UVR might exert persistent effects on a subset of genes, which may serve as UV gene expression signature with a biomarker potential for assessing UVR-induced skin damages. To identify UV-induced DEGs with biomarker potential, we performed bioinformatics and statistical analyses on DEGs derived from 30 mJ/cm^2^ UVR exposure to isolate DEGs that were conserved among different donors (N0, N1, N2, and N6). We identified 401 conserved UV signature DEGs, which we designated as the putative UV biomarker panel (S4 Table). To test protein-protein interactions (PPIs) among the protein products of these UVSGs, we performed STRING (Search Tool for the Retrieval of Interacting Genes/Proteins) network analysis using the Pajek software (version 3.1) [28] based on the known and predicted protein interactions available in the STRING database (version 10) [29]. A STRING cutoff score of 0.7 was used to select PPIs with high confidence. Altogether, we found 54 vertices (genes) and 106 edges (interactions) among the UVSGs (Fig 4A). Clustering analysis using the VOS algorithm [30] to maximize modularity within each cluster revealed 11 modules that were connected with each other except the histone protein cluster (Fig 4A). Among the UVSGs, 13 of them showed more than 5 interacting neighbors (degree), also known as the hubs on the PPI network, including IL6 (19), PTGS2 (15), and IL1B (12), highlighting the potentially central roles of these genes in mediating UVR responses.

### Dysregulation of UVSGs in human SCCs

Compelling evidence suggests that UVR is the main etiological factor in SCC pathogenesis [4, 31]. To test whether UVSGs were dysregulated in human SCCs, we performed similar RNA-Seq analyses to identify DEGs in human SCCs by comparing the transcriptome of the SCC tumor tissue with that of the adjacent normal skin from the same patient. We then performed GSEA to determine the enrichment of the UVSGs in the SCC DEGs, or vice versa. As shown in Fig 4B and 4C, GSEA analyses revealed a significant mutual enrichment between the UVSG and the SCC signature (p = 0.006 and 0.02, respectively). When we used an SCC signature discovered by microarray-based analyses [32], we observed a significant enrichment between the SCC signature and the UVSG as well (p = 5.19e-05 by Fisher exact test analysis), confirming the molecular similarities between the UVSG and SCC signature.

To test whether the identified UVSG is specific for skin cancer, we performed additional GSEA analyses to compare the UVSG with gene sets dysregulated in 14 other human cancer types (obtained from the TCGA RNA-Seq database). Each cancer type contained at least 6 pairs of primary tumors and adjacent normal tissues (Table 1). We generated DEG sets specific for each cancer type using paired t-test. Each resulting cancer DEG set was then used in GSEA analyses to assess the mutual enrichment between the UVSG and each cancer signature. As summarized in Table 1, there was no significant enrichment between the UVSG and other cancer signatures (p>0.05) except for thyroid cancer (p = 0.0222, Table 1). The similarity between the UVSG and thyroid cancer signature might be related to the fact that ionizing radiation is a significant risk factor for thyroid cancer [33], and that UVR and other radiations may share common target genes involved in pathways such as DNA damage and inflammation. A recent prospective study also found a non-linear association between UVR and thyroid cancer [34]. Further studies are needed to determine whether UVR may indeed increase thyroid cancer risk.

**Table 1:** Summary of the GSEA results between UVSGs and gene sets dysregulated in different human cancers derived from the TCGA database.

Cancer tissue origin	# of matched tumor/normal samples	NES of tumor gene set on UVR signature genes	NES of UVR signature genes on tumor gene set	Average NES	p-value (lower T = F)
**Bladder**	19	-2.96	-1.93	-2.445	0.993
**Breast**	110	-3.11	-1.43	-2.27	0.988
**Colon**	41	-1.47	-0.762	-1.116	0.868
**Head & Neck**	40	-1.87	-1.46	-1.665	0.952
**Kidney (renal)**	72	1.64	1.47	1.555	0.06
**Kidney (papillary)**	32	-0.999	-1.22	-1.1095	0.866
**Liver**	50	-3.19	-2.17	-2.68	0.996
**Lung (adeno)**	57	-2.54	-1.41	-1.975	0.976
**lung (squamous)**	50	-3.29	-1.96	-2.625	0.996
**Prostate**	52	-1.42	-1.44	-1.43	0.924
**Rectal**	8	-0.944	-1.08	-1.012	0.844
**Stomach**	29	-1.85	0.356	-0.747	0.772
**Thyroid** **	59	2.17	1.85	2.01	0.0222
**Uterine**	23	-3.35	-1.1	-2.225	0.987

NES: normalized enrichment score;

** indicates p-value < 0.05

To test the stability of the UV gene expression signature following exposure, we performed RNA-Seq on keratinocytes exposed to 30 mJ/cm^2^ of UV to generate a UV-induced DEG list at Day 21 after exposure. Cross-comparison of the UVSG with the Day 21 DEG list revealed an overlap of 144 genes (Fig 4D and S5 Table) (p<2.2e-16 per Fisher's exact test), suggesting that a significant portion of the UVSGs maintained their initial UV responsiveness long after exposure. Furthermore, cross-comparison of the UVSG derived in keratinocytes and the SCC signature (consisting of DEGs between the SCC and adjacent normal skin tissues) revealed that the UVSGs were significantly enriched in the SCC signatures (p<2.2e-16 per Fisher's exact test) (Fig 4E and 4F), underscoring the potential of the UVSGs as biomarkers for assessing UVR damage and predicting skin cancer risk.

### Role of UVSGs in skin cancer cell proliferation and viability

Project Achilles leverages both biological and computational analyses to identify genes that affect cancer cell survival and/or proliferation using a genome-wide shRNA library screening in over 200 cancer cell lines [27]. Based on the degree of depletion of a specific shRNA-treated cancer cell population following infection, a depletion score is assigned to each shRNA. The depletion score is therefore inversely correlated with the role of its target gene in cancer cell survival, based on the assumption that loss of a key cancer survival gene (due to RNAi effect) is detrimental to the infected cells [27]. Given that Achilles data were derived from loss-of-function analysis, we focused on 67 UVSGs that were upregulated in both SCCs and by UVR (FC>2). We have validated 25 of the 67 genes in the Achilles database in multiple cancer cells lines. We queried the Achilles database with these 25 genes to determine which are critical to skin cancer cell proliferation and/or survival. Using the Wilcoxon test, we determined that 11 out of the 25 genes had significantly lower depletion scores in skin cancer cell lines compared to other non-skin cancer lines (p<0.05, Table 2), indicating that this subset of UVSGs may play key roles in skin carcinogenesis. The depletion scores of the shRNAs targeting these 11 genes in 5 skin cancer cell lines, together with the median depletion scores of the same shRNAs in non-skin cancer lines, and the p-values from Wilcoxon tests are summarized in Table 2. These analyses suggest that a subset of UVSGs may be targeted in future skin cancer prevention and therapeutic development.

**Table 2:** Summary of the UVSGs critical for skin cancer cell survival.

Genes	Skin vs. other cancer lines	DS of skin cancer cell lines					DS of other cancer cell lines
	Wilcoxon. Test (p-value)	A2058	C32	COLO741	HS944T	SKMEL5	Median
SLPI	0.00141	-1.4	-1.33	-1.07	-0.875	-1.68	-0.599
KLK7	0.00468	-0.311	-0.375	-0.962	-0.706	-0.93	0.0862
KRT13	0.00621	-0.439	-0.159	-1.18	0.0826	-0.877	0.54
NHLH2	0.00933	-0.882	-1.78	-0.864	-0.98	-1.63	-0.449
GPRC5A	0.0106	-1.53	-1.78	-2.63	-1.29	-1.63	-0.963
HIST1H2BK	0.0167	-1.3	-0.579	-1.02	-1.44	-0.65	-0.361
IGFBP3	0.017	0.277	-1.22	0.332	0.677	0.286	0.765
SPOCD1	0.022	-0.833	-1.45	-0.861	-0.639	-1.21	-0.342
IFI27	0.0273	-0.0243	-2.92	-1.26	-1.13	-2.22	-0.528
KLK11	0.0286	-1.14	-1.63	-0.566	-0.914	-1.07	-0.577
TNFSF4	0.0374	-1.35	-2.3	-1.87	-1.33	-1.3	-1.17

DS: Depletion Scores

## Discussion

Despite decades of research, no consensus panel of molecular biomarkers is available for accurate assessment of UVR damage and stratification of skin cancer risk. mRNA transcripts have been successfully used as molecular biomarkers to enable early detection and diagnosis of disease and to track disease progression. To facilitate the development of a biomarker-based sensitive method to assess UVR damage and stratify skin cancer risk, we employed RNA-Seq to identify UV-induced gene expression signatures to establish a UV biomarker panel. Utilizing bioinformatics and statistical analyses, we compiled a UV biomarker panel consisting of 401 genes that are consistently altered by UVR and conserved among keratinocytes from different donors. We further demonstrate that alterations in the mRNA expression of the UVSGs persisted 21 days after exposure, illustrating the stability and reliability of the identified UV biomarker genes in future diagnostic applications. The persistent gene expression changes may be due to UVR-induced epigenetic changes in DNA methylation and/or histone modifications at target gene loci following UVR. The dose-dependent response among some of the UVSGs further suggests that this novel UV biomarker panel may offer quantitative assessments of UVR skin damage and thus help identify individuals at increased levels of risk for developing skin cancer.

While some of the UVR target genes uncovered in this study have been reported in previous studies [14–17], we have identified many new UVR target genes due to the near-complete coverage of the transcriptome by RNA-Seq compared to the limits of previous microarray-based analyses. Our experimental design also allows comprehensive characterizations of the UV-responsive kinetics in the keratinocyte transcriptome (Figs 2 and 3). We would like to point out, however, that we performed only one RNA-Seq for each cell line at each UVR dose or time point. Furthermore, our findings are based on cultured cells, which do not involve the complex interactions between keratinocytes and other cell types *in vivo*. Therefore, additional validations of the identified UVSGs are warranted in future studies. Among the DDR genes down-regulated by UVR (Fig 1B), many of them encode DNA helicases, DNA-dependent ATPases, and pyrophosphatases that participate in DNA replication and repair (S6 Table). DDR plays pivotal roles not only in maintaining genome integrity and stability, but it also has a broad impact on cell cycle regulation, chromatin dynamics, and apoptosis [35]. This may explain the observations that these related pathways all appear as the top UV-responsive pathways (Fig 1B). Among the genes upregulated by UVR exposure in the inflammatory response pathway, HDAC5 and HADC9 are two epigenetic regulators belonging to the class IIA family of histone deacetylases that are known to regulate adaptive immunity [36], underscoring the importance of epigenetic regulation of UVR-induced skin immune response.

The UV biomarker panel identified in this study consists of more genes (401) than other biomarker panels currently used in clinical diagnosis of various diseases [37–39]. A large biomarker panel can offer better coverage and accuracy in assessing UV impact and skin cancer risk. New molecular tests can be developed based on the UV biomarker genes to replace the current MED-based method in testing the UV-protective efficacy of sunscreen products. The significant similarity between the UV signature and SCC signature suggests that such biomarker-based tests may also enable clinical diagnosis and skin cancer risk prediction in susceptible individuals following severe sunburns. Due to the steep decreases in the run times and costs of next-generation sequencing technologies, profiling an individual’s transcriptome has become a feasible clinical undertaking. We anticipate that the UV biomarker panel, together with ever-improving RNA-Seq and bioinformatics tools, will greatly facilitate the development of next-generation diagnostic tests to enhance skin cancer prevention and early detection to reduce the incidence of this prevalent human malignancy.

## Supporting Information

S1 Table Keratinocyte lines and experimental UVR conditions (DOCX)

S2 Table Genes displaying time-dependent changes in mRNA expression following UVR. (DOCX)

S3 Table Genes displaying dose-dependent changes in mRNA expression following UVR. (DOCX)

S4 Table Conserved UVR signature genes in response to 30mJ/cm^2^ UVR. (DOCX)

S5 Table Overlapping genes between UVR transcriptomic gene set and the DEG set from 21 days after UVR. (DOCX)

S6 Table Top UVR-responsive pathways and associated UVR target genes. (XLSX)

## References

[1] Guy GP Jr, Machlin SR, Ekwueme DU, Yabroff KR. Prevalence and costs of skin cancer treatment in the U.S., 2002–2006 and 2007–2011. American journal of preventive medicine. 2015;48(2):183–7. 10.1016/j.amepre.2014.08.036 25442229 PMC4603424

[2] Rogers HW, Weinstock MA, Feldman SR, Coldiron BM. Incidence Estimate of Nonmelanoma Skin Cancer (Keratinocyte Carcinomas) in the US Population, 2012. JAMA dermatology. 2015;151(10):1081–6. 10.1001/jamadermatol.2015.1187 . 25928283

[3] The Surgeon General's Call to Action to Prevent Skin Cancer. Reports of the Surgeon General. Washington (DC)2014. 25320835

[4] Wu S, Han J, Laden F, Qureshi AA. Long-term ultraviolet flux, other potential risk factors, and skin cancer risk: a cohort study. Cancer epidemiology, biomarkers & prevention: a publication of the American Association for Cancer Research, cosponsored by the American Society of Preventive Oncology. 2014;23(6):1080–9. 10.1158/1055-9965.EPI-13-0821 24876226 PMC4151553

[5] Robinson JK. Sun exposure, sun protection, and vitamin D. Jama. 2005;294(12):1541–3. . 16193624 10.1001/jama.294.12.1541

[6] Pleasance ED, Cheetham RK, Stephens PJ, McBride DJ, Humphray SJ, Greenman CD, et al A comprehensive catalogue of somatic mutations from a human cancer genome. Nature. 2010;463(7278):191–6. 10.1038/nature08658 20016485 PMC3145108

[7] Lautenschlager S, Wulf HC, Pittelkow MR. Photoprotection. Lancet. 2007;370(9586):528–37. 10.1016/S0140-6736(07)60638-2 . 17693182

[8] Osterwalder U, Herzog B. Sun protection factors: world wide confusion. The British journal of dermatology. 2009;161 Suppl 3:13–24. 10.1111/j.1365-2133.2009.09506.x . 19775352

[9] Bens G. Sunscreens. Advances in experimental medicine and biology. 2014;810:429–63. . 25207381 10.1007/978-1-4939-0437-2_25

[10] Dennis LK, Beane Freeman LE, VanBeek MJ. Sunscreen use and the risk for melanoma: a quantitative review. Annals of internal medicine. 2003;139(12):966–78. . 14678916 10.7326/0003-4819-139-12-200312160-00006

[11] Hacker E, Boyce Z, Kimlin MG, Wockner L, Pollak T, Vaartjes SA, et al The effect of MC1R variants and sunscreen on the response of human melanocytes in vivo to ultraviolet radiation and implications for melanoma. Pigment cell & melanoma research. 2013;26(6):835–44. 10.1111/pcmr.12157 . 23962207

[12] Seite S, Fourtanier A, Moyal D, Young AR. Photodamage to human skin by suberythemal exposure to solar ultraviolet radiation can be attenuated by sunscreens: a review. The British journal of dermatology. 2010;163(5):903–14. 10.1111/j.1365-2133.2010.10018.x . 20977441

[13] Heckman CJ, Chandler R, Kloss JD, Benson A, Rooney D, Munshi T, et al Minimal Erythema Dose (MED) testing. Journal of visualized experiments: JoVE. 2013;(75):e50175 10.3791/50175 23748556 PMC3734971

[14] Dawes JM, Antunes-Martins A, Perkins JR, Paterson KJ, Sisignano M, Schmid R, et al Genome-wide transcriptional profiling of skin and dorsal root ganglia after ultraviolet-B-induced inflammation. PloS one. 2014;9(4):e93338 10.1371/journal.pone.0093338 24732968 PMC3986071

[15] de la Fuente H, Lamana A, Mittelbrunn M, Perez-Gala S, Gonzalez S, Garcia-Diez A, et al Identification of genes responsive to solar simulated UV radiation in human monocyte-derived dendritic cells. PloS one. 2009;4(8):e6735 10.1371/journal.pone.0006735 19707549 PMC2727914

[16] Yang G, Zhang G, Pittelkow MR, Ramoni M, Tsao H. Expression profiling of UVB response in melanocytes identifies a set of p53-target genes. The Journal of investigative dermatology. 2006;126(11):2490–506. 10.1038/sj.jid.5700470 . 16888633

[17] Rieger KE, Chu G. Portrait of transcriptional responses to ultraviolet and ionizing radiation in human cells. Nucleic acids research. 2004;32(16):4786–803. 10.1093/nar/gkh783 15356296 PMC519099

[18] Dazard JE, Gal H, Amariglio N, Rechavi G, Domany E, Givol D. Genome-wide comparison of human keratinocyte and squamous cell carcinoma responses to UVB irradiation: implications for skin and epithelial cancer. Oncogene. 2003;22(19):2993–3006. 10.1038/sj.onc.1206537 . 12771951

[19] Takao J, Ariizumi K, Dougherty II, Cruz PD Jr. Genomic scale analysis of the human keratinocyte response to broad-band ultraviolet-B irradiation. Photodermatology, photoimmunology & photomedicine. 2002;18(1):5–13. . 11982916 10.1034/j.1600-0781.2002.180102.x

[20] Kadekaro AL, Leachman S, Kavanagh RJ, Swope V, Cassidy P, Supp D, et al Melanocortin 1 receptor genotype: an important determinant of the damage response of melanocytes to ultraviolet radiation. FASEB journal: official publication of the Federation of American Societies for Experimental Biology. 2010;24(10):3850–60. 10.1096/fj.10-158485 20519635 PMC3229421

[21] Tripp CS, Blomme EA, Chinn KS, Hardy MM, LaCelle P, Pentland AP. Epidermal COX-2 induction following ultraviolet irradiation: suggested mechanism for the role of COX-2 inhibition in photoprotection. The Journal of investigative dermatology. 2003;121(4):853–61. 10.1046/j.1523-1747.2003.12495.x . 14632205

[22] Sun X, Kim A, Nakatani M, Shen Y, Liu L. Distinctive molecular responses to ultraviolet radiation between keratinocytes and melanocytes. Experimental dermatology. 2016 10.1111/exd.13057 . 27119462 PMC5295856

[23] Anders S, Huber W. Differential expression analysis for sequence count data. Genome biology. 2010;11(10):R106 10.1186/gb-2010-11-10-r106 20979621 PMC3218662

[24] Gentleman RC, Carey VJ, Bates DM, Bolstad B, Dettling M, Dudoit S, et al Bioconductor: open software development for computational biology and bioinformatics. Genome biology. 2004;5(10):R80 10.1186/gb-2004-5-10-r80 15461798 PMC545600

[25] Huang da W, Sherman BT, Lempicki RA. Systematic and integrative analysis of large gene lists using DAVID bioinformatics resources. Nature protocols. 2009;4(1):44–57. 10.1038/nprot.2008.211 . 19131956

[26] Subramanian A, Tamayo P, Mootha VK, Mukherjee S, Ebert BL, Gillette MA, et al Gene set enrichment analysis: a knowledge-based approach for interpreting genome-wide expression profiles. Proceedings of the National Academy of Sciences of the United States of America. 2005;102(43):15545–50. 10.1073/pnas.0506580102 16199517 PMC1239896

[27] Cowley GS, Weir BA, Vazquez F, Tamayo P, Scott JA, Rusin S, et al Parallel genome-scale loss of function screens in 216 cancer cell lines for the identification of context-specific genetic dependencies. Scientific data. 2014;1:140035 10.1038/sdata.2014.35 25984343 PMC4432652

[28] Batagelj V, Mrvar A. Pajek—Analysis and visualization of large networks. Math Visual. 2004:77–103. WOS:000186345600004.

[29] Szklarczyk D, Franceschini A, Wyder S, Forslund K, Heller D, Huerta-Cepas J, et al STRING v10: protein-protein interaction networks, integrated over the tree of life. Nucleic acids research. 2015;43(Database issue):D447–52. 10.1093/nar/gku1003 25352553 PMC4383874

[30] van Eck NJ, Waltman L, Dekker R, van den Berg J. A Comparison of Two Techniques for Bibliometric Mapping: Multidimensional Scaling and VOS. J Am Soc Inf Sci Tec. 2010;61(12):2405–16. 10.1002/asi.21421. WOS:000284231100003.

[31] Fartasch M, Diepgen TL, Schmitt J, Drexler H. The Relationship Between Occupational Sun Exposure and Non-Melanoma Skin Cancer. Dtsch Arztebl Int. 2012;109(43):715–U14. 10.3238/arztebl.2012.0715. WOS:000311722200001. 23181135 PMC3498471

[32] Hudson LG, Gale JM, Padilla RS, Pickett G, Alexander BE, Wang J, et al Microarray analysis of cutaneous squamous cell carcinomas reveals enhanced expression of epidermal differentiation complex genes. Molecular carcinogenesis. 2010;49(7):619–29. 10.1002/mc.20636 20564339 PMC3626076

[33] Boice JD Jr. Radiation-induced thyroid cancer—what's new? Journal of the National Cancer Institute. 2005;97(10):703–5. 10.1093/jnci/dji151 . 15900034

[34] Lin SW, Wheeler DC, Park Y, Cahoon EK, Hollenbeck AR, Freedman DM, et al Prospective study of ultraviolet radiation exposure and risk of cancer in the United States. International journal of cancer Journal international du cancer. 2012;131(6):E1015–23. 10.1002/ijc.27619 22539073 PMC3402606

[35] Harper JW, Elledge SJ. The DNA damage response: ten years after. Molecular cell. 2007;28(5):739–45. 10.1016/j.molcel.2007.11.015 . 18082599

[36] Falkenberg KJ, Johnstone RW. Histone deacetylases and their inhibitors in cancer, neurological diseases and immune disorders. Nature reviews Drug discovery. 2014;13(9):673–91. 10.1038/nrd4360 . 25131830

[37] You YN, Rustin RB, Sullivan JD. Oncotype DX((R)) colon cancer assay for prediction of recurrence risk in patients with stage II and III colon cancer: A review of the evidence. Surgical oncology. 2015;24(2):61–6. 10.1016/j.suronc.2015.02.001 . 25770397

[38] Zanotti L, Bottini A, Rossi C, Generali D, Cappelletti MR. Diagnostic tests based on gene expression profile in breast cancer: from background to clinical use. Tumour biology: the journal of the International Society for Oncodevelopmental Biology and Medicine. 2014;35(9):8461–70. 10.1007/s13277-014-2366-2 . 25048969

[39] Gyorffy B, Hatzis C, Sanft T, Hofstatter E, Aktas B, Pusztai L. Multigene prognostic tests in breast cancer: past, present, future. Breast cancer research: BCR. 2015;17:11 10.1186/s13058-015-0514-2 25848861 PMC4307898

