# Supplementary figures and images for: Transcriptome Analysis Identifies the Dysregulation of Ultraviolet Target Genes in Human Skin Cancers
Source: PLoS One. 2016 Sep 19;11(9):e0163054. doi: 10.1371/journal.pone.0163054 (PMC5028058; doi:10.1371/journal.pone.0163054)

**S1Table.** Keratinocyte lines and experimental UVR conditions

**
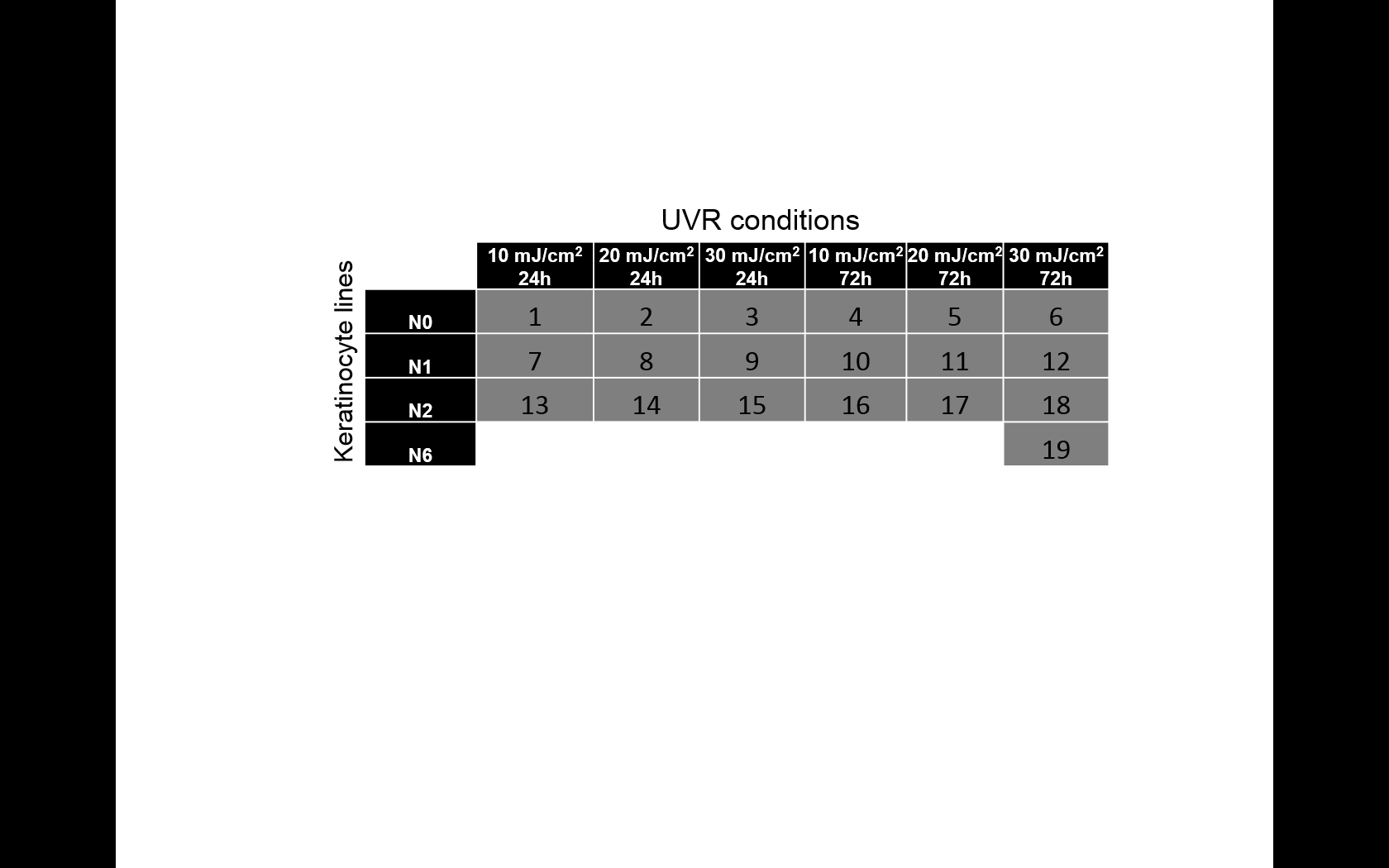
**

Supplement: S1 Table — (DOCX) [file pone.0163054.s001.docx]
