# Supplementary material for: Transcriptome Analysis Identifies the Dysregulation of Ultraviolet Target Genes in Human Skin Cancers
Source: PLoS One. 2016 Sep 19;11(9):e0163054. doi: 10.1371/journal.pone.0163054 (PMC5028058; doi:10.1371/journal.pone.0163054)
Supplement: S2 Table — (DOCX) [file pone.0163054.s002.docx]

**S2 Table.** Genes displaying time-dependent changes in mRNA expression following UVR

|  |  |  |  | Down-regulated |  |  |  |  |
| --- | --- | --- | --- | --- | --- | --- | --- | --- |
| ANLN |  | CENPH |  | GINS1 |  | MND1 |  | SGOL1 |
| ARHGAP11A |  | CENPI |  | GINS2 |  | MTBP |  | SGOL2 |
| ARHGAP11B |  | CENPJ |  | GINS4 |  | MYBL1 |  | SHCBP1 |
| ASF1B |  | CENPK |  | GLT8D2 |  | MYBL2 |  | SKA1 |
| ASPM |  | CENPM |  | GPR63 |  | MYH15 |  | SKA3 |
| ATAD2 |  | CENPN |  | GSG2 |  | NCAPG |  | SKP2 |
| ATAD5 |  | CENPO |  | GTSE1 |  | NCAPG2 |  | SLC43A3 |
| AURKA |  | CENPW |  | H2AFX |  | NCAPH |  | SLFN13 |
| AURKB |  | CEP55 |  | HAUS8 |  | NDC80 |  | SMC2 |
| BARD1 |  | CHAF1A |  | HELLS |  | NEIL3 |  | SMC4 |
| BIRC5 |  | CHEK1 |  | HIST1H2BH |  | NEK2 |  | SNORD17 |
| BLM |  | CHRNA5 |  | HJURP |  | NRGN |  | SNORD28 |
| BORA |  | CKAP2L |  | HMGB2 |  | NUF2 |  | SOX11 |
| BRCA1 |  | CKS1B |  | HMMR |  | NUSAP1 |  | SPAG5 |
| BRCA2 |  | CLSPN |  | HPDL |  | OIP5 |  | SPC24 |
| BRIP1 |  | DBF4 |  | HYOU1 |  | ORC1 |  | SPC25 |
| BUB1 |  | DBF4B |  | IL7R |  | ORC6 |  | STAMBPL1 |
| BUB1B |  | DDX12P |  | INCENP |  | PALMD |  | STIL |
| C11orf82 |  | DEPDC1 |  | IQGAP3 |  | PBK |  | SULT1E1 |
| C14orf80 |  | DHFR |  | KIAA0101 |  | PCDH18 |  | SUV39H1 |
| C15orf42 |  | DLEU1 |  | KIAA1524 |  | PCDHAC2 |  | TACC3 |
| C16orf59 |  | DLGAP5 |  | KIF11 |  | PEG10 |  | TCF19 |
| C1orf112 |  | DSCC1 |  | KIF14 |  | PFAS |  | TGM4 |
| C9orf100 |  | DTL |  | KIF15 |  | PKMYT1 |  | THBS1 |
| CASC5 |  | DUSP9 |  | KIF18A |  | PLK1 |  | TIMELESS |
| CCDC150 |  | E2F1 |  | KIF18B |  | PLK4 |  | TK1 |
| CCNA2 |  | E2F8 |  | KIF20A |  | POLE2 |  | TMEM97 |
| CCNB1 |  | EDNRA |  | KIF20B |  | POLQ |  | TOP2A |
| CCNB2 |  | EME1 |  | KIF23 |  | POLR3G |  | TPX2 |
| CCNE2 |  | ERCC6L |  | KIF24 |  | PRC1 |  | TRAIP |
| CCNF |  | ESCO2 |  | KIF2C |  | PRIM1 |  | TRIM59 |
| CDC20 |  | ESPL1 |  | KIF4A |  | PSMC3IP |  | TRIP13 |
| CDC25A |  | EXO1 |  | KIF4B |  | PTGS1 |  | TROAP |
| CDC25C |  | FAM111B |  | KIFC1 |  | RACGAP1 |  | TTK |
| CDC45 |  | FAM167A |  | KPNA2 |  | RAD51 |  | UBE2C |
| CDC6 |  | FAM54A |  | LBR |  | RAD51AP1 |  | UBE2S |
| CDC7 |  | FAM64A |  | LMNB1 |  | RAD54B |  | UHRF1 |
| CDCA2 |  | FAM72A |  | LOC100128191 |  | RAD54L |  | UTP20 |
| CDCA3 |  | FAM72B |  | LOC100506711 |  | RBL1 |  | WDR4 |
| CDCA5 |  | FAM83D |  | MAD2L1 |  | RECQL4 |  | WDR62 |
| CDCA8 |  | FANCA |  | MCM10 |  | RFC3 |  | WDR65 |
| CDK1 |  | FANCB |  | MCM3 |  | RGMB |  | WDR76 |
| CDKN2C |  | FANCD2 |  | MCM5 |  | RMI1 |  | XRCC2 |
| CDKN3 |  | FANCI |  | MCM7 |  | RRM2 |  | ZNF367 |
| CDT1 |  | FBXO5 |  | MELK |  | S1PR1 |  | ZNF492 |
| CENPA |  | FEN1 |  | MKI67 |  | SEMA3D |  | ZNF724P |
| CENPE |  | FKBP11 |  | MMP2 |  | SFTA1P |  | ZWINT |
| CENPF |  | FOXM1 |  | MMS22L |  | SFXN2 |  |  |

|  |  | Up-regulated |  |  |
| --- | --- | --- | --- | --- |
| ABCA12 |  | GAMT |  | NOTCH3 |
| ABLIM3 |  | GDA |  | NUPR1 |
| ACBD4 |  | GGT1 |  | PAPL |
| ACTA2 |  | GIPR |  | PIK3IP1 |
| ADAMTS13 |  | GPNMB |  | PLA2G4C |
| ADAMTS7 |  | GRIN3B |  | PLEKHG1 |
| ADAMTSL4 |  | HBEGF |  | PNLIPRP3 |
| ADHFE1 |  | HIST1H2AC |  | ProSAPiP1 |
| ADSSL1 |  | HLA-G |  | PRSS22 |
| AIFM3 |  | HSD17B14 |  | PRSS8 |
| ALDH3B2 |  | HSD17B2 |  | PSORS1C1 |
| ALOX15B |  | ICAM1 |  | QPCT |
| ANKRD29 |  | ICAM4 |  | REEP6 |
| APOE |  | IDUA |  | RET |
| ATP12A |  | IL32 |  | RHBDL1 |
| B3GALT4 |  | IL33 |  | RNF208 |
| BLNK |  | IRAK2 |  | RUNDC3A |
| BNIP3L |  | IRF5 |  | RYR1 |
| BTBD19 |  | ITIH4 |  | S100A4 |
| C11orf35 |  | ITPKC |  | S100A6 |
| C19orf46 |  | KCTD11 |  | SAA1 |
| C1orf126 |  | KIAA1370 |  | SGPP2 |
| C1orf38 |  | KLHDC9 |  | SIRPB2 |
| C1orf88 |  | KLHL24 |  | SLC28A3 |
| C5orf41 |  | KLK11 |  | SLPI |
| C6orf138 |  | KRT15 |  | SORT1 |
| C7orf10 |  | KRT19 |  | SPNS2 |
| C9orf7 |  | KRT23 |  | SULT1A1 |
| CARD14 |  | KRT37 |  | TCP11L2 |
| CBX7 |  | KYNU |  | TIMP2 |
| CCDC64B |  | LCN2 |  | TLCD2 |
| CCL20 |  | LINC00086 |  | TLR2 |
| CD68 |  | LOC100049716 |  | TM7SF2 |
| CLDN7 |  | LOC100129781 |  | TMEM38A |
| COX6B2 |  | LOC100131096 |  | TMEM61 |
| CRCT1 |  | LOC100131564 |  | TMEM91 |
| CST6 |  | LOC100505623 |  | TMPRSS4 |
| CTSS |  | LOC100507452 |  | TNFAIP8L3 |
| CUL9 |  | LOC284080 |  | TNFRSF14 |
| CYGB |  | LOC284440 |  | TREM2 |
| DKFZp434J0226 |  | LOC646471 |  | TSPAN10 |
| DPP4 |  | LOC728975 |  | TTC39A |
| DQX1 |  | LYNX1 |  | TTC9 |
| DYRK1B |  | MEG3 |  | TTLL3 |
| EPHB2 |  | MIR21 |  | TXNIP |
| ESPN |  | MLPH |  | VAMP5 |
| FBXO32 |  | MME |  | VNN1 |
| FGF11 |  | MNT |  | WFDC5 |
| FN3K |  | MUC1 |  | YPEL2 |
| FOLR3 |  | MXD4 |  | YPEL3 |
| FTH1 |  | MXI1 |  | YPEL4 |
| FTL |  | MYO15B |  | ZFHX2 |
| FXYD3 |  | N4BP2L1 |  | ZNF185 |
| G0S2 |  | NDRG4 |  | ZNF610 |
| GABARAPL1 |  | NFATC4 |  |  |
