# Supplementary material for: Transcriptome Analysis Identifies the Dysregulation of Ultraviolet Target Genes in Human Skin Cancers
Source: PLoS One. 2016 Sep 19;11(9):e0163054. doi: 10.1371/journal.pone.0163054 (PMC5028058; doi:10.1371/journal.pone.0163054)
Supplement: S3 Table — (DOCX) [file pone.0163054.s003.docx]

**S3 Table.** Genes displaying dose-dependent changes in mRNA expression following UVR

|  |  |  |  | Down-regulated |  |  |  |  |
| --- | --- | --- | --- | --- | --- | --- | --- | --- |
| ABCC4 |  | CEP112 |  | FKBP11 |  | MCM10 |  | RUNDC3B |
| ABI3BP |  | CEP128 |  | FOXM1 |  | MCM3 |  | RYR3 |
| ADAMTSL1 |  | CEP55 |  | GALNT10 |  | MCM5 |  | SCAPER |
| AGTPBP1 |  | CHAF1A |  | GALNTL4 |  | MCM6 |  | SCFD2 |
| AKAP6 |  | CHEK1 |  | GINS1 |  | MCM7 |  | SCLT1 |
| AKAP7 |  | CHRNA5 |  | GINS2 |  | MELK |  | SCMH1 |
| ALDH1L2 |  | CHSY3 |  | GINS4 |  | METAP1D |  | SCN9A |
| ALG14 |  | CIT |  | GIPC2 |  | MGC16121 |  | SDF2L1 |
| ALMS1 |  | CKAP2L |  | GLI1 |  | MKI67 |  | SDK1 |
| ANKRD44 |  | CKS1B |  | GLT8D2 |  | MMP2 |  | SEMA3D |
| ANLN |  | CLMP |  | GMDS |  | MMS22L |  | SEMA3E |
| ANXA6 |  | CLSPN |  | GNB3 |  | MND1 |  | SEMA5A |
| APLN |  | CNTLN |  | GPC6 |  | MOXD1 |  | SERGEF |
| ARHGAP11A |  | CNTN1 |  | GPHN |  | MSRA |  | SFXN2 |
| ARHGAP11B |  | COL12A1 |  | GPR39 |  | MTBP |  | SGOL1 |
| ARHGAP19 |  | COL18A1 |  | GPR63 |  | MYBL2 |  | SGOL2 |
| ARHGAP33 |  | COL4A1 |  | GPSM2 |  | NCAPD2 |  | SHCBP1 |
| ARSB |  | COL4A2 |  | GRB14 |  | NCAPG |  | SKA1 |
| ASF1B |  | COL8A1 |  | GRIA1 |  | NCAPG2 |  | SKA3 |
| ASNS |  | COMMD1 |  | GRIP1 |  | NCAPH |  | SKP2 |
| ASPM |  | COMMD10 |  | GSG2 |  | NCKAP5 |  | SLC16A9 |
| ATAD2 |  | CPS1 |  | GTDC1 |  | NCOA1 |  | SLC2A13 |
| ATAD5 |  | CREB5 |  | GTSE1 |  | NDC80 |  | SLC43A1 |
| AURKA |  | CTNNAL1 |  | H2AFX |  | NEIL3 |  | SLC7A5 |
| AURKB |  | DBF4 |  | HAUS8 |  | NEK2 |  | SLC8A1 |
| B3GALTL |  | DBF4B |  | HELLS |  | NTM |  | SLFN13 |
| BARD1 |  | DCHS1 |  | HJURP |  | NUF2 |  | SLIT3 |
| BBS9 |  | DDX12P |  | HMCN1 |  | NUSAP1 |  | SMC2 |
| BCAT1 |  | DEPDC1 |  | HMGB2 |  | ODZ3 |  | SMC4 |
| BCL2 |  | DEPDC1B |  | HMGCS1 |  | ORC1 |  | SMYD3 |
| BEND6 |  | DHFR |  | HMMR |  | ORC6 |  | SNORA51 |
| BIRC5 |  | DIAPH2 |  | HS6ST2 |  | OSBPL6 |  | SNX29 |
| BLM |  | DIAPH3 |  | INCENP |  | OXCT1 |  | SPAG17 |
| BORA |  | DLEU1 |  | IQCK |  | P4HA3 |  | SPAG5 |
| BRCA1 |  | DLEU2 |  | IQGAP3 |  | PALM2 |  | SPATA13 |
| BRCA2 |  | DLGAP5 |  | ISPD |  | PBK |  | SPATA17 |
| BRIP1 |  | DLL1 |  | ITGA1 |  | PCDHAC2 |  | SPATA5 |
| BUB1 |  | DMC1 |  | ITGA4 |  | PDE4D |  | SPATA6 |
| BUB1B |  | DNAH5 |  | ITPR1 |  | PDGFC |  | SPC24 |
| C11orf82 |  | DOCK10 |  | ITPR2 |  | PEG10 |  | SPC25 |
| C12orf26 |  | DPYD |  | KCNK10 |  | PFAS |  | SPEF2 |
| C12orf48 |  | DPYSL3 |  | KCNQ5 |  | PHGDH |  | STAG1 |
| C14orf80 |  | DRP2 |  | KHDRBS3 |  | PID1 |  | STIL |
| C15orf42 |  | DSCC1 |  | KIAA0825 |  | PIF1 |  | STK33 |
| C16orf59 |  | DTL |  | KIAA1524 |  | PIK3C2G |  | STS |
| C21orf58 |  | DTWD2 |  | KIF11 |  | PKI55 |  | STXBP4 |
| C3orf26 |  | DYNC2H1 |  | KIF14 |  | PKMYT1 |  | SUPT3H |
| C4orf21 |  | DZIP3 |  | KIF15 |  | PLK1 |  | SUV39H1 |
| C5 |  | E2F1 |  | KIF18A |  | PLK4 |  | SYT1 |
| C9orf100 |  | E2F8 |  | KIF18B |  | PLXNC1 |  | TACC3 |
| C9orf93 |  | EDA |  | KIF20A |  | PLXND1 |  | TBX1 |
| CADPS2 |  | EFCAB11 |  | KIF20B |  | POLA1 |  | TCF19 |
| CAMKMT |  | EFCAB2 |  | KIF23 |  | POLE2 |  | TDRD9 |
| CASC2 |  | EFHC2 |  | KIF24 |  | POLQ |  | THBS1 |
| CASC5 |  | ELAVL2 |  | KIF26B |  | POLR3G |  | THBS2 |
| CBS |  | ELOVL6 |  | KIF2C |  | PRC1 |  | TIMELESS |
| CCDC150 |  | ELP4 |  | KIF4A |  | PRDM5 |  | TK1 |
| CCDC152 |  | EME1 |  | KIF4B |  | PRICKLE1 |  | TLL1 |
| CCDC18 |  | ENOX1 |  | KIFC1 |  | PRIM1 |  | TLR6 |
| CCDC3 |  | EPB41L2 |  | KLHL13 |  | PRKCA |  | TMEM97 |
| CCNA2 |  | ERCC6L |  | KNTC1 |  | PRR11 |  | TMTC2 |
| CCNB1 |  | ESPL1 |  | KPNA2 |  | PRTFDC1 |  | TNC |
| CCNB2 |  | EXO1 |  | L3MBTL4 |  | PRUNE2 |  | TNS1 |
| CCNF |  | EXTL2 |  | LARGE |  | PSAT1 |  | TOP2A |
| CDC20 |  | FAF1 |  | LBR |  | PSMC3IP |  | TPK1 |
| CDC25C |  | FAM111B |  | LEF1 |  | PSRC1 |  | TPX2 |
| CDC45 |  | FAM167A |  | LFNG |  | PTGS1 |  | TRAIP |
| CDC6 |  | FAM172A |  | LHFP |  | PTPRG |  | TRAPPC9 |
| CDC7 |  | FAM54A |  | LMCD1 |  | PTPRZ1 |  | TRIM59 |
| CDCA2 |  | FAM64A |  | LMNB1 |  | PYCR1 |  | TRIP13 |
| CDCA3 |  | FAM72A |  | LNP1 |  | RACGAP1 |  | TROAP |
| CDCA5 |  | FAM72B |  | LOC100128191 |  | RAD51 |  | TTC26 |
| CDCA7 |  | FAM72D |  | LOC100288637 |  | RAD51AP1 |  | TTK |
| CDCA8 |  | FAM83D |  | LOC100506711 |  | RAD51B |  | TUBA1C |
| CDH4 |  | FANCA |  | LOC100506994 |  | RAD54B |  | TYW1B |
| CDK1 |  | FANCB |  | LOC100507552 |  | RAD54L |  | UBE2C |
| CDKAL1 |  | FANCC |  | LOC100652789 |  | RANBP17 |  | UHRF1 |
| CDKN3 |  | FANCD2 |  | LOC642846 |  | RAPGEF4 |  | USP13 |
| CDON |  | FANCI |  | LOC647946 |  | RBL1 |  | UTP20 |
| CDT1 |  | FAR2 |  | LRIG1 |  | RECQL4 |  | WDPCP |
| CENPA |  | FARS2 |  | LRP8 |  | RFC3 |  | WDR4 |
| CENPE |  | FBN2 |  | LRRC6 |  | RMI1 |  | WDR62 |
| CENPF |  | FBXL17 |  | LRRIQ1 |  | RNLS |  | WDR65 |
| CENPH |  | FBXL7 |  | LTBP1 |  | ROBO1 |  | WDR7 |
| CENPI |  | FBXO43 |  | LZTS1 |  | ROR1 |  | WDR76 |
| CENPJ |  | FBXO5 |  | MAD2L1 |  | RPL22L1 |  | WHSC1 |
| CENPM |  | FGFBP1 |  | MAGI3 |  | RPS6KA2 |  | WWOX |
| CENPN |  | FGGY |  | MAP6 |  | RRM2 |  | XRCC2 |
| CENPO |  | FHIT |  | MATN3 |  | RSRC1 |  | XRCC4 |
| CENPW |  | FIGN |  | MBOAT1 |  | RUNDC2A |  | XYLT1 |
|  |  |  |  |  |  | ZNF730 |  | ZNF367 |

|  |  | Up-regulated |  |  |
| --- | --- | --- | --- | --- |
| A4GALT |  | GGT6 |  | NLRP10 |
| ABCD1 |  | GIPR |  | NR1D1 |
| ABHD4 |  | GJB4 |  | NR4A1 |
| ABLIM3 |  | GLRX |  | OCLN |
| ACAP1 |  | GLS2 |  | P4HTM |
| ACER2 |  | GPR172B |  | PAPL |
| ADCK3 |  | GPR37 |  | PCDH1 |
| AIM1L |  | GPRASP1 |  | PDE6B |
| AKR1B10 |  | GPRC5A |  | PGPEP1 |
| AKR1C1 |  | GRB7 |  | PHLDB3 |
| AKR1C2 |  | GREB1 |  | PI3 |
| ARHGAP30 |  | GRHL3 |  | PIDD |
| ARNT2 |  | GSDMA |  | PLAUR |
| ATF3 |  | H1F0 |  | PLEKHG6 |
| AVPI1 |  | HAP1 |  | PNLIPRP3 |
| B3GNT3 |  | HAPLN3 |  | PNMAL1 |
| BCL2L1 |  | HBEGF |  | PNRC1 |
| BCL6 |  | HBP1 |  | PPP1R15A |
| BIK |  | HCAR2 |  | PPP1R3B |
| BIRC3 |  | HCAR3 |  | PRDM1 |
| BMF |  | HDAC5 |  | PRICKLE4 |
| BNIP3L |  | HDAC9 |  | ProSAPiP1 |
| BTBD19 |  | HEPHL1 |  | PRSS22 |
| BTG1 |  | HES2 |  | PRSS8 |
| C11orf35 |  | HIST1H1C |  | PTGS2 |
| C11orf9 |  | HIST1H2AC |  | PVRL4 |
| C16orf5 |  | HIST1H2BD |  | QPCT |
| C17orf103 |  | HIST1H2BK |  | RAB11FIP1 |
| C1orf51 |  | HIST2H2BE |  | RASSF5 |
| C1orf74 |  | HIST3H2A |  | REEP6 |
| C5orf41 |  | HLA-G |  | RET |
| C9orf7 |  | HMOX1 |  | RGAG4 |
| CARD18 |  | HSD17B14 |  | RGS16 |
| CASP9 |  | HSD3B7 |  | RGS2 |
| CCDC11 |  | HSPB8 |  | RHCG |
| CCK |  | ICAM1 |  | RHPN1 |
| CD55 |  | ID2 |  | RNASE7 |
| CD68 |  | IL1B |  | RND2 |
| CD74 |  | IL1RN |  | RORA |
| CDKN1A |  | IL23A |  | RRAD |
| CDKN2B |  | IL36RN |  | RRM2B |
| CDKN2D |  | IL8 |  | RUNDC3A |
| CDSN |  | INPP5J |  | S100A4 |
| CGN |  | IRAK2 |  | SALL4 |
| CHST2 |  | IRF5 |  | SAMD10 |
| CITED2 |  | IRF6 |  | SBK1 |
| CLCF1 |  | ISG20 |  | SCNN1A |
| CLDN1 |  | ISYNA1 |  | SELPLG |
| CLDN23 |  | ITPKC |  | SEMA3B |
| CLDN4 |  | KCNN4 |  | SERPINB1 |
| CLDN7 |  | KCTD11 |  | SERPINB2 |
| CLEC2B |  | KIAA1257 |  | SERTAD1 |
| CLU |  | KIAA1370 |  | SESN1 |
| CNFN |  | KLHL24 |  | SLAMF7 |
| CRB3 |  | KLK10 |  | SLC46A1 |
| CRCT1 |  | KLK11 |  | SLPI |
| CRISPLD2 |  | KLRG2 |  | SMOC1 |
| CRYAB |  | KRT13 |  | SPRR1B |
| CSF1 |  | KRT15 |  | SPRR3 |
| CST6 |  | KRT19 |  | SQSTM1 |
| CTSS |  | KRT34 |  | SYNGR3 |
| CYP2S1 |  | KRT37 |  | TCP11L2 |
| DAPK1 |  | KRT7 |  | THBD |
| DBNDD1 |  | KRT80 |  | TLR2 |
| DEFB1 |  | LACC1 |  | TM7SF2 |
| DENND1C |  | LBH |  | TMEM125 |
| DHDH |  | LCE1B |  | TMEM184A |
| DHRS3 |  | LCE1C |  | TMEM27 |
| DKFZp434J0226 |  | LCN2 |  | TMEM61 |
| DPP4 |  | LIF |  | TMPRSS13 |
| DUSP10 |  | LOC100133190 |  | TNFAIP2 |
| DYRK1B |  | LOC100505974 |  | TNFRSF10C |
| EDA2R |  | LOC100506377 |  | TNFRSF14 |
| ENO2 |  | LOC100506746 |  | TOB1 |
| ENTPD3 |  | LOC100507429 |  | TP53INP1 |
| ERBB3 |  | LOC100507452 |  | TP53INP2 |
| FAM131C |  | LOC151475 |  | TPPP |
| FAM43A |  | LOC441869 |  | TRAF1 |
| FAM46A |  | LOC728975 |  | TRAF3IP3 |
| FAM84A |  | LYPD5 |  | TRIM17 |
| FAM86HP |  | MAP1LC3A |  | TSPAN10 |
| FDXR |  | MCHR1 |  | TTC9 |
| FLJ32255 |  | MDM2 |  | UCA1 |
| FLJ43663 |  | MEG3 |  | ULBP1 |
| FLNC |  | MUC1 |  | ULK1 |
| FN3K |  | MXD1 |  | VAMP5 |
| FOLR3 |  | MXD4 |  | VNN1 |
| FTH1 |  | MYBPHL |  | VWCE |
| FTL |  | MYH16 |  | YPEL3 |
| FUT2 |  | NCF2 |  | YPEL4 |
| FUT3 |  | NDRG4 |  | ZFYVE1 |
| G0S2 |  | NEAT1 |  | ZNF425 |
| GDA |  | NFKBIA |  | ZNF432 |
| GDF15 |  | NFKBIZ |  | ZNF610 |
| GGT1 |  | NIPAL4 |  | ZNF702P |
