# Supplementary material for: Transcriptome Analysis Identifies the Dysregulation of Ultraviolet Target Genes in Human Skin Cancers
Source: PLoS One. 2016 Sep 19;11(9):e0163054. doi: 10.1371/journal.pone.0163054 (PMC5028058; doi:10.1371/journal.pone.0163054)
Supplement: S4 Table — (DOCX) [file pone.0163054.s004.docx]

**S4 Table.** Conserved UVR signature genes in response to 30mJ/cm^2^ UVR

| ABCD1 |  | DUSP13 |  | KISS1 |  | MIR23A |  | SCN3B |
| --- | --- | --- | --- | --- | --- | --- | --- | --- |
| ABLIM3 |  | ELF3 |  | KLHL34 |  | MIR29A |  | SCNN1B |
| ADAMTS14 |  | ENKUR |  | KLK10 |  | MIR614 |  | SCNN1D |
| AGAP11 |  | ENTPD3 |  | KLK14 |  | MME |  | SCNN1G |
| ALG1L |  | EPHB2 |  | KLK6 |  | MMP1 |  | SEMA3B |
| ALOX5 |  | ESM1 |  | KPNA7 |  | MMP3 |  | SERPINB1 |
| ANGPTL4 |  | FA2H |  | KPRP |  | MSH4 |  | SERPINB2 |
| ANKRD20A5P |  | FAM110C |  | KRT13 |  | MSX1 |  | SHBG |
| ANKRD29 |  | FAM115C |  | KRT19 |  | MUC20 |  | SHC2 |
| ANKRD33 |  | FAM167B |  | KRT23 |  | MUC3A |  | SHC4 |
| ANKRD56 |  | FAM182B |  | KRT34 |  | MUM1L1 |  | SIGLEC15 |
| APOBEC3H |  | FAM25A |  | KRT37 |  | MYBPHL |  | SLAMF7 |
| ARC |  | FAM46C |  | KRT38 |  | MYH16 |  | SLC22A14 |
| ARHGAP30 |  | FAM65C |  | KRT4 |  | MYO7A |  | SLC25A41 |
| ARNT2 |  | FAM83E |  | KRT7 |  | MYPN |  | SLC25A45 |
| ASPRV1 |  | FBP1 |  | KRT78 |  | NCCRP1 |  | SLC40A1 |
| ATG9B |  | FER1L4 |  | KRT80 |  | NCF4 |  | SLC44A4 |
| B3GNT3 |  | FLJ34208 |  | KRT81 |  | NDRG4 |  | SLC6A14 |
| BCAN |  | FLJ43663 |  | KRTAP19-1 |  | NFE2 |  | SLC6A20 |
| BCL2L1 |  | FLNC |  | KYNU |  | NKAIN4 |  | SLC6A9 |
| BMF |  | FOXA1 |  | LBH |  | NKD2 |  | SLC7A11 |
| BMP7 |  | FTL |  | LCE1A |  | NLGN3 |  | SLCO2A1 |
| C10orf10 |  | FUT2 |  | LCE1B |  | OCLN |  | SLPI |
| C14orf34 |  | FUT3 |  | LCE1D |  | OXER1 |  | SMOC1 |
| C15orf48 |  | GABBR2 |  | LCE1E |  | PADI1 |  | SNORD119 |
| C15orf52 |  | GAD1 |  | LCE1F |  | PAPL |  | SNX32 |
| C17orf28 |  | GAS7 |  | LCE2A |  | PCDH1 |  | SOD3 |
| C17orf67 |  | GAST |  | LCE3A |  | PCDHAC1 |  | SPNS2 |
| C1orf228 |  | GCKR |  | LCE3D |  | PCDHGB8P |  | SPP1 |
| C1orf68 |  | GDA |  | LCE3E |  | PDE4C |  | SPRR2B |
| C20orf195 |  | GDF15 |  | LCE6A |  | PDE9A |  | SPRR2G |
| C2orf54 |  | GDNF |  | LCN2 |  | PDGFRA |  | SPRR3 |
| C3orf25 |  | GEM |  | LDB3 |  | PIK3R5 |  | SPRR4 |
| C6orf15 |  | GGT1 |  | LEMD1 |  | PKD2L2 |  | STC2 |
| C7orf10 |  | GJB4 |  | LGI2 |  | PLA2G10 |  | STRC |
| CAMP |  | GLRX |  | LIF |  | PLA2G16 |  | STX16-NPEPL1 |
| CAPN12 |  | GOLT1A |  | LINC00086 |  | PLA2G2F |  | SULT1A1 |
| CARD18 |  | GPR172B |  | LINC00303 |  | PLA2G4C |  | SULT1A2 |
| CASKIN1 |  | GPRC5A |  | LOC100049716 |  | PLAC8L1 |  | SYNPO2L |
| CATSPERG |  | GREB1 |  | LOC100128342 |  | PLEKHB1 |  | SYT5 |
| CCDC110 |  | GRIN3B |  | LOC100129617 |  | PNLIPRP3 |  | TCTEX1D4 |
| CCDC62 |  | GRIP2 |  | LOC100130331 |  | PNMAL1 |  | TIMP2 |
| CCIN |  | HAP1 |  | LOC100287036 |  | POLD4 |  | TJP3 |
| CD68 |  | HCAR3 |  | LOC100287082 |  | POSTN |  | TLCD2 |
| CD70 |  | HIST1H1C |  | LOC100289251 |  | POU4F1 |  | TM4SF19 |
| CD8A |  | HIST1H2AC |  | LOC100505623 |  | PRPS1L1 |  | TMEM125 |
| CDH16 |  | HIST1H2AE |  | LOC100505639 |  | PRR9 |  | TMEM22 |
| CDKN1A |  | HIST1H2BC |  | LOC100505710 |  | PRSS22 |  | TMEM38A |
| CDKN2D |  | HIST1H2BD |  | LOC100505974 |  | PRSS27 |  | TMEM40 |
| CDSN |  | HIST1H2BG |  | LOC100505994 |  | PSCA |  | TMEM88 |
| CEACAM1 |  | HIST1H3D |  | LOC100506328 |  | PSG2 |  | TMIE |
| CEACAM6 |  | HIST1H4H |  | LOC100506377 |  | PSG6 |  | TMPRSS11B |
| CELF5 |  | HIST2H2BE |  | LOC100506411 |  | PSG7 |  | TMPRSS11E |
| CHRNA9 |  | HIST2H2BF |  | LOC100506801 |  | PTCH2 |  | TMPRSS13 |
| CLCF1 |  | HLA-G |  | LOC100506810 |  | PTGS2 |  | TNFAIP2 |
| CLDN17 |  | HMOX1 |  | LOC100507025 |  | PTPN22 |  | TNFRSF10C |
| CLDN23 |  | HPGD |  | LOC100507065 |  | PVRL4 |  | TNXB |
| CLDN4 |  | HRASLS |  | LOC100507140 |  | RAB6B |  | TP53INP2 |
| CLDN7 |  | HSD17B14 |  | LOC100507145 |  | RASSF5 |  | TREML1 |
| CLDN9 |  | HSD17B2 |  | LOC100507452 |  | REN |  | TRIM63 |
| CLEC18B |  | HSPB8 |  | LOC100653024 |  | RET |  | TRPV3 |
| CLEC3B |  | ICAM1 |  | LOC145757 |  | RGS16 |  | TSPAN1 |
| CLGN |  | IGFL1 |  | LOC151475 |  | RNASE7 |  | TTC9 |
| CLIC6 |  | IGFN1 |  | LOC152225 |  | RNF182 |  | UCA1 |
| CNFN |  | IGSF22 |  | LOC284080 |  | RNF222 |  | UPK2 |
| CRCT1 |  | IL13RA2 |  | LOC284804 |  | RNF223 |  | USP2 |
| CRISPLD2 |  | IL1B |  | LOC285095 |  | RNF224 |  | USP44 |
| CRYM |  | IL1RL1 |  | LOC388282 |  | RPLP0P2 |  | VAV1 |
| CSF3 |  | IL23A |  | LOC440993 |  | RPTN |  | VNN1 |
| CST6 |  | IL36B |  | LOC643401 |  | RRAD |  | VWCE |
| CT62 |  | IL6 |  | LOC646329 |  | RRAGD |  | ZEB2 |
| CTSL3 |  | IL8 |  | LOC692247 |  | RUNDC3A |  | ZMYND15 |
| CYGB |  | ILDR1 |  | LOC728741 |  | S100A12 |  | ZNF425 |
| CYP24A1 |  | INSC |  | LOC728975 |  | S100A5 |  | ZP4 |
| CYP4F3 |  | KC6 |  | LRRC4 |  | S100A6 |  | ZPLD1 |
| CYTH4 |  | KCNG1 |  | LYPD5 |  | S100A7 |  | ZSCAN1 |
| DAPK1 |  | KCNN4 |  | MAP1LC3A |  | S100P |  | ZSCAN4 |
| DEFB1 |  | KHDC1L |  | MARCO |  | SALL4 |  |  |
| DHRS9 |  | KIAA1239 |  | MCHR1 |  | SCARF1 |  |  |
| DKK4 |  | KIAA1683 |  | MEOX1 |  | SCARNA16 |  |  |
| DPP4 |  | KIF26A |  | MESP1 |  | SCG2 |  |  |
