# Supplementary material for: Transcriptome Analysis Identifies the Dysregulation of Ultraviolet Target Genes in Human Skin Cancers
Source: PLoS One. 2016 Sep 19;11(9):e0163054. doi: 10.1371/journal.pone.0163054 (PMC5028058; doi:10.1371/journal.pone.0163054)
Supplement: S5 Table — (DOCX) [file pone.0163054.s005.docx]

**S5 Table.** Overlapping genes between UVR transcriptomic gene set and the DEG set from 21 days after UVR

| ADAMTS14 |  | LOC646329 |  | MCHR1 |
| --- | --- | --- | --- | --- |
| ANGPTL4 |  | HIST1H2AC |  | MIR23A |
| ANKRD20A5P |  | HIST1H2AE |  | MME |
| ANKRD56 |  | HIST1H2BC |  | MMP1 |
| ARHGAP30 |  | HIST1H2BD |  | MMP3 |
| ATG9B |  | HIST1H2BG |  | MYPN |
| BCAN |  | HIST1H3D |  | OCLN |
| C10orf10 |  | HIST1H4H |  | PADI1 |
| C14orf34 |  | HIST2H2BE |  | PCDHAC1 |
| C15orf52 |  | HIST2H2BF |  | PKD2L2 |
| C1orf68 |  | HMOX1 |  | PLAC8L1 |
| C20orf195 |  | HSD17B2 |  | POSTN |
| C7orf10 |  | ICAM1 |  | PRSS22 |
| CAPN12 |  | IL13RA2 |  | PSCA |
| CCDC62 |  | IL1RL1 |  | PTCH2 |
| CD8A |  | IL23A |  | PTGS2 |
| CEACAM1 |  | IL6 |  | PVRL4 |
| CLDN4 |  | IL8 |  | RGS16 |
| CLDN7 |  | KIAA1239 |  | RPLP0P2 |
| CLEC18B |  | KLK10 |  | RRAD |
| CLEC3B |  | KPRP |  | S100A12 |
| CRCT1 |  | KRT13 |  | S100A7 |
| CRYM |  | KRT19 |  | SCARF1 |
| CSF3 |  | KRT23 |  | SCARNA16 |
| CST6 |  | KRT7 |  | SCNN1D |
| CT62 |  | KRTAP19-1 |  | SHC2 |
| CYGB |  | KYNU |  | SHC4 |
| DEFB1 |  | LBH |  | SLAMF7 |
| DPP4 |  | LCE1F |  | SLC22A14 |
| ENTPD3 |  | LCE3D |  | SLC44A4 |
| FER1L4 |  | LCN2 |  | SPNS2 |
| FLJ43663 |  | LEMD1 |  | SPP1 |
| FLNC |  | LGI2 |  | STX16-NPEPL1 |
| FUT3 |  | LIF |  | SULT1A2 |
| GAD1 |  | LINC00086 |  | TM4SF19 |
| GDA |  | LOC100129617 |  | TMEM88 |
| GLRX |  | LOC100130331 |  | TMPRSS11E |
| GOLT1A |  | LOC100287036 |  | TMPRSS13 |
| GPR172B |  | LOC100506377 |  | TNXB |
| GRIP2 |  | LOC100506801 |  | TREML1 |
| HCAR3 |  | LOC100507025 |  | VNN1 |
| LOC152225 |  | LOC100507452 |  |  |
